# The Insurance Bill

**Published:** 1911-06-03

**Authors:** Francis H. Moore

**Affiliations:** General Superintendent and Secretary, Royal Infirmary, Liverpool.


Dear Sir,—Mr. Carnt's excellent article on the probable effect on our voluntary hospitals of the State Insurance Bill as it has been drafted leaves little to be added.

We must "wait and see" what modifications will be brought about by the protests being organised throughout the country; but it is clear that the Bill must hasten the day when other means of raising funds besides purely voluntary contributions will have to be adopted.

If it is to be State or Municipal aid, it might be done, as it is in America and elsewhere, without alienating the voluntary giver.—Yours faithfully,

Francis H. Moore,

General Superintendent and Secretary,

May 29. Royal Infirmary, Liverpool.

